# The evaluation of dietary protein levels and the comparative application of the NASEM model for ration formulation in finishing Nellore cattle

**DOI:** 10.1007/s11250-026-05428-5

**Published:** 2026-11-02

**Authors:** Kaique de Souza Nascimento, Lorena Emily de Lemos Mota Bomfim, Victor Rezende Moreira Couto, Mauricia Brandão da Silva, Ana Laura Araujo Lopes, Terezinha Teixeira de Souza, Marcos Vinicius de Castro Ferraz, Juliano Jose de Resende Fernandes

**Affiliations:** 1 Escola de veterinária e Zootecnia, Universidade Federal de Goiás, Goiânia, Goiás 74690- 900 Brasil; 2 Instituto de Ciências Sociais, Educação e Zootecnia, Universidade Federal do Amazonas, Parintins, Amazonas 69152240 Brasil

**Keywords:** Energy, NASEM, Mechanistic, Microorganisms, Recycling, Tropical livestock nutrition

## Abstract

Typically, true protein sources are the most expensive ingredients in feed formulations for feedlot cattle. However, low-protein diets can limit animal performance. This study aimed to use the mechanistic NASEM model to formulate diets with similar metabolizable protein levels while varying crude protein content and evaluating the impact of these adjustments on performance and nutrient digestibility in feedlot Nellore bulls. A total of 120 Nellore bulls (initial body weight = 386.18 ± 23 kg; 24 ± 1 months of age) were allocated to 15 pens (8 bulls/pen). A randomized complete block design was used, with bulls stratified into five blocks according to their initial body weight. Within each block, pens were randomly assigned to one of three dietary treatments differing in crude protein (CP) concentration: High CP (HCP), Medium CP (MCP), and Low CP (LCP), while maintaining the same metabolizable protein (MP) level across all diets. The CP and MP concentrations were as follows: HCP: 140 g CP/ kg dry matter (DM) and 86 g MP/kg DM; MCP: 125 g CP/kg DM and 86 g MP/kg DM; LCP: 110 g CP/kg DM and 86 g MP/kg DM. Final BW (*P* < 0.001) and ADG (*P* = 0.003) as well as dry matter intake (DMI) (*P* < 0.001) increased linearly with higher dietary CP concentrations. Similarly, both hot and cold carcass weights (*P* = 0.002) and carcass gain (*P* < 0.001) increased linearly with increasing CP levels in the diets. Comparing the NASEM model predictions with observed data, the predicted DMI from the model was approximately 15% and 10% lower than the observed values in the 14% and 12.5% CP treatments, respectively. Additionally, the estimated DMI by NASEM was 3% higher than the observed intake in 11% of CP. In summary, increasing dietary CP from 11% to 14% by replacing starch with true protein increased DMI and improved the performance of feedlot Nellore bulls. The mechanistic NASEM model underestimated DMI in diets containing 12.5% and 14% CP, which led to lower predictions of MP, energy balance, and ADG in feedlot Nellore bulls.

## Introduction

The primary goal in feedlot operations should be maximizing financial and productive efficiency by adjusting diets in terms of energy and protein to enhance microbial protein production and reduce environmental nitrogen excretion. Among the ingredients used in feedlot diet formulation, protein sources are the most expensive. Optimizing dietary protein inclusion is crucial since low levels can limit animal performance, while excessive levels may reduce efficiency due to the energy cost associated with nitrogen excretion (Hales et al. 2013).

Research shows that balancing protein on diet may increases the efficiency of nitrogen capture by rumen microbes, reducing nitrogen losses as urea and ammonia (Vasconcelos and Galyean 2007). Over supplying RDP or using low-quality protein sources results in elevated nitrogen excretion and greater environmental impact, including ammonia volatilization and nitrous oxide emissions (Coelho et al. 2022). According to research by Silvestre and Millen (2021), diets formulated by Brazilian nutritionists contain, on average, 13.7% crude protein (CP), ranging from 12% to 15.8%. One way to evaluate feed formulation and optimize results is by using nutrient requirement tables such as the Beef Cattle Nutrient Requirements Model (NASEM 2016) and Valadares et al. (2016). On average, these models recommend lower CP levels than those commonly used in Brazilian feedlots, ranging between 11% (NASEM 2016) and 12.5% (Valadares et al. 2016) for Nellore cattle. The higher CP concentrations found in Brazilian diets are often due to the need for safety margins, as nutritionists account for the significant variability of feed ingredients available in the country and the generally low operational efficiency.

The NASEM (2016) proposes two models—empirical and mechanistic—for calculating energy and protein requirements and predictions. The empirical model, like its predecessors, utilizes tabulated values of total digestible nutrients (TDN) for each ingredient to estimate microbial protein synthesis (MPmic), rumen-degradable protein (RDP) requirements, and tabulated values of rumen-undegradable protein (RUP) to determine metabolizable protein (MP). Following the NASEM standard, the 2016 model also includes a mechanistic approach, which considers the degradation kinetics of carbohydrates and proteins to determine TDN, MPmic, RUP, and ultimately, MP availability in the intestine (NASEM 2016; Valadares et al. 2016). The choice between models should be based on the amount of available data regarding both diet composition and animal characteristics. While the mechanistic model is more precise, it requires more detailed input information.

Therefore, this study aimed to evaluate the mechanistic model of NASEM (2016) to formulate diets with similar metabolizable protein levels while varying crude protein content and assessing the impact of these adjustments on performance and nutrient digestibility in feedlot Nellore cattle.

## Materials and methods

The experimental procedures were approved by the Animal Ethics Committee of the Federal University of Goiás, Brazil (protocol no. 050/19).

### Animals, facilities, and experimental design

A total of 120 Nellore bulls (initial body weight [BW] = 386.18 ± 23 kg; 24 ± 1 months of age) were allocated to 15 collective pens of 77 m² (7.7 × 10 m; 8 bulls/pen), equipped with concrete feed bunks (7.7 m of linear space) and water troughs (500 L per two pens). The animals were blocked by BW into five blocks. A randomized complete block design was used, in which animals were blocked by initial BW into five blocks. The experimental unit was the pen, consisting of eight animals, with five replicates per treatment. All animals were individually identified with an unique ear tag, received a topical cypermethrin (Cypermil Pour-on; Ourofino Saúde Animal; Cravinhos, SP, Brazil), and were treated for external and internal parasites with albendazole (Ricobendazole 10; Ouro Fino Saúde Animal).

### Experimental period and adaptation protocol

The experimental period consisted of 14 days of adaptation to the facilities and diet, followed by 105 days of experimental period. The adaptation phase was performed as a step-up protocol, divided into two periods of 7 d each. The management consisted in reducing the inclusion of sugarcane bagasse by 10% every 7 d, whereas the diet offered during the first and second 7-d period contained (dry matter [DM] basis) 30% and 20% of sugarcane bagasse, respectively. The bulls were weighed at the beginning and end of the trial after a 14-hour fasting period (solid feed only).

### Diets and experimental treatments

The treatments consisted of three diets with different crude protein (CP) levels: High CP (HCP; 140 g CP/kg DM), Medium CP (MCP; 125 g CP/kg DM), and Low CP (LCP; 110 g CP/kg DM). However, all diets contained the same metabolizable protein (MP) level (86 g MP/kg DM). The MP values for each diet were determined based on NASEM formulas, considering the model’s updated nitrogen (N) recycling estimates (Table 1).

**Table 1:** Ingredients used in the final diets and their chemical composition

Ingredients (g/kg DM)	Treatment^1^		
	HCP	MCP	LCP
Sugarcane bagasse	100	101	101
Cracked corn	740	800	846
Soybean meal	134	69	21
Urea	5	9	11
Mineral mix^2^	21	21	21
Chemical composition (g/kg DM)			
DM (g/kg as fed)	641.8	661.4	647.1
Organic Matter	969.9	954.8	961.4
Ether extract	33.2	34.1	34.9
Neutral Detergent Fiber	252.8	236.6	258.2
Ash	30.1	45.2	38.6
Crude protein	139.9	125.9	113.1
Rumen degradable protein²	100,7	91,1	80,9
Bacterial MP²	60.73	61.44	61.96
Feed MP²	25.77	24.63	24.23
Total MP²	86.51	86.07	86.26
Starch²	505.6	545.7	576.5
NEm (Mcal/kg DM)²	1.96	1.96	1.96
NEg (Mcal/kg DM)²	1.31	1.31	1.32
Predict DMI (kg/day)²	8.84	8.88	8.87

¹ HCP: High Crude Protein; MCP: Medium Crude Protein; LCP: Low Crude Protein. DM: Dry matter; MP: Metabolizable Protein; NEm: Net Energy Net for maintenance; NEg: Net Energy for gain

² Mineral supplement (Ganho 60; Ganho Animal Nutrition, Goiânia – GO): Guaranteed levels per kg:

Calcium: 185 g; Phosphorus: 60 g; Sodium: 111 g; Magnesium: 10 g; Sulfur: 18 g;

Copper: 1000 mg; Manganese: 800 mg; Zinc: 3600 mg; Cobalt: 75 mg; Iodine: 75 mg;

Selenium: 11 mg; Fluorine: 600 mg; 1200 mg monensin (Rumensin, Elanco Animal Health, São Paulo – SP)

³ Values derived from the 8th revised edition of the Nutrient Requirements of Beef Cattle (NASEM, 2016)

Observed DMI and ADG were not used as inputs in the NASEM model. These variables were generated by the model based on the input animal characteristics and diet composition and were subsequently compared with the observed experimental values.

The dietary composition is presented in Table 1. The diets were formulated to maintain the same net energy for gain (1.31 Mcal/kg DM) and MP levels (86 g/kg DM), following NASEM equations, including N recycling estimates. Expected variations in CP and starch content were observed due to the dietary formulations.

### Feeding management and intake evaluation

The animals were fed once daily at 08:00 AM. The diets were provided ad libitum, adjusted to 105% of the previous day’s intake, and composed of sugarcane bagasse (100 g/kg DM) as the roughage source and concentrate (900 g/kg DM; Table 1). Feed refusals were collected, weighed, sampled daily, and stored at -20 °C for subsequent chemical analysis. Dry matter intake (DMI) was determined by calculating the difference between the feed offered and refusals, both expressed in DM. Weekly intake was monitored within each treatment to assess treatment × time interactions, and the average DMI for each week was recorded.

### Nutrient digestibility, chemical analyses and energy calculations

Apparent nutrient digestibility was assessed by collecting fecal samples immediately after defecation over three consecutive days (days 60–62), with an average 12-hour interval between collections. Samples were collected from at least six animals per pen, homogenized, and pooled into six composite samples per pen. After three days, 500 g of each composite sample was frozen at -20 °C for further analysis, along with feed samples. Fecal excretion was estimated using the indigestible neutral detergent fiber (iNDF) marker (INCT-CA F-009/1 method).

Energy gain (EG; Mcal/d) was estimated using the equation:

EG = (0.0557 × BW^0.075^) × GMD^1.097^, where BW is body weight (kg).

Maintenance energy (ME; Mcal/d) was estimated as:

ME = 0.077 × BW^0.75^.

Net energy for maintenance (NEm) and gain (NEg) were calculated as follows:

NEm = (-b-((b²)-(4ac))^0.5^))/2, where a = -0.41× ME, b = 0.877 × ME + 0.41 × DMI + EG e c = -0.877 × DMI; NEg = 0.877 × DMI.

### Slaughter procedures

The animals were slaughtered in a commercial slaughterhouse. Immediately after slaughter, the hot carcass weight (kg) was recorded to calculate carcass yield relative to final body weight. Cold carcass weight was determined after 24 h of chilling at 2 ± 2 °C. Bromatological analysis included the determination of dry matter (DM; INCT-CA G-001/1 method), crude protein (CP; INCT-CA N-001/1 method), ether extract (EE; INCT-CA G-004/1 method), ash (ASH; INCT-CA M-001/1 method), organic matter (OM), and neutral detergent fiber (NDF; INCT-CA F-002/1 method).

### Statistical analysis

All data were tested for normality using the Shapiro-Wilk test, and variance homogeneity was assessed using Levene’s test before ANOVA. Discrepant data (outliers identified as studentized residuals > 3 or < − 3) were removed from the analyses, which resulted in the exclusion of one bull from the MCP treatment. The variance analysis was performed using the GLM procedure in SAS (SAS Institute Inc., Cary, NC). The effects of CP levels (treatments) in the diets were evaluated using linear (L) and quadratic (Q) orthogonal contrasts. Effects were considered significant at *P* < 0.05, while trends were for *P* > 0.05 and *P* < 0.10.

## Results

Nutrient intake increased linearly as dietary CP concentration increased (Table 2). Dry matter intake rose from 8.58 kg/d in LCP to 9.90 kg/d in MCP and 10.21 kg/d in HCP (*P* < 0.001). Crude protein intake increased substantially from 0.98 kg/d in LCP to 1.21 kg/d in MCP and 1.42 kg/d in HCP (*P* < 0.001). Similarly, NDF intake increased from 2.23 to 2.36 to 2.59 kg/d (*P* = 0.003). Non-fibrous carbohydrate intake increased from 4.75 kg/d (LCP) to 5.55 kg/d (MCP) and 5.57 kg/d (HCP) (*P* < 0.001).

**Table 2:** Effect of treatments in the nutrient intake and digestibility of Nellore bulls

Variables	Treatment^1^			SEM	*P*-Valor		
	HCP	MCP	LCP		Treatment	Linear	Quadratic
Intake							
Dry Matter (kg/d)	10.21	9.90	8.58	0.212	0.001	< 0.001	0.087
Organic Matter (kg/d)	9.91	9.46	8.26	0.007	0.001	< 0.001	0.169
Crude Protein (kg/d)	1.42	1.21	0.98	0.027	< 0.001	< 0.001	0.642
Neutral Detergent Fiber (kg/d)	2.59	2.36	2.23	0.059	0.008	0.003	0.533
Ether Extract (kg/d)	0.34	0.34	0.30	0.007	0.004	0.003	0.038
Non-fibrous Carbohydrate (kg/d)	5.57	5.55	4.75	0.111	0.001	< 0.001	0.021
Digestibility (g/kg DM)							
Dry Matter	607.45	699.17	772.41	24.544	< 0.001	0.001	0.766
Organic Matter	640.25	716.13	788.49	24.048	0.001	0.002	0.954
Crude Protein	562.62	648.57	709.83	29.893	0.008	0.008	0.745
Neutral Detergent Fiber	468.85	584.82	675.40	27.833	< 0.001	< 0.001	0.719
Ether Extract	775.62	823.43	859.35	14.767	0.003	0.004	0.749
Non-fibrous Carbohydrate	731.76	780.22	853.23	24.111	0.005	0.007	0.688

^1^ HCP: High Crude Protein; MCP: Medium Crude Protein; LCP: Low Crude Protein

In contrast to intake, nutrient digestibility decreased linearly with increasing CP levels. Dry matter digestibility decreased from 772 g/kg in LCP to 699 g/kg in MCP and 607 g/kg in HCP (*P* = 0.001). Crude protein digestibility decreased from 710 g/kg in LCP to 649 g/kg in MCP and 563 g/kg in HCP (*P* = 0.008). Fiber digestion was also decreased linearly, with NDF digestibility falling sharply from 675 to 585 to 469 g/kg (*P* < 0.001). Non-fibrous carbohydrate digestibility also decreased linearly (853, 780, and 732 g/kg; *P* = 0.007).

**Fig. 1:**
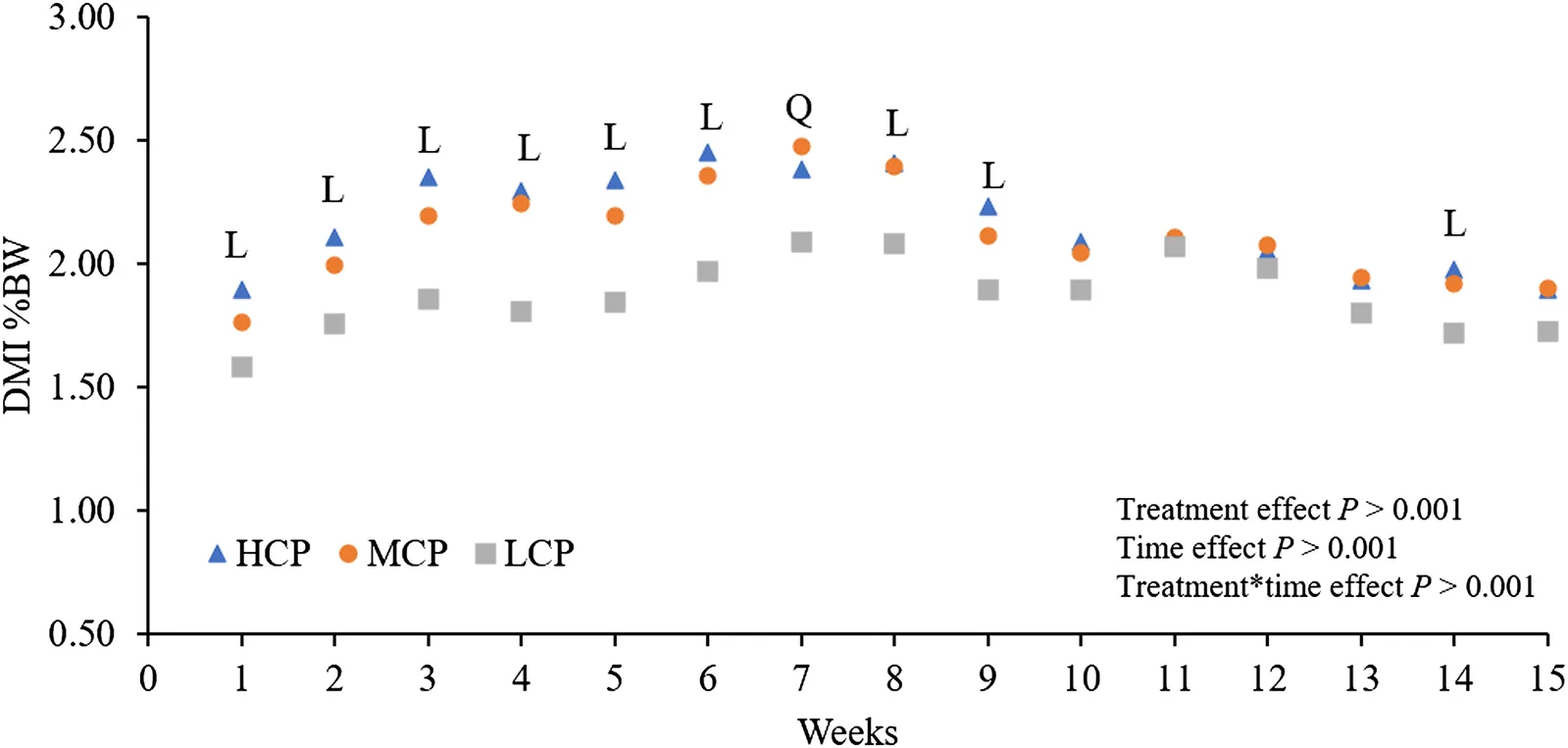
Day matter intake as a percentage of BW over the experimental weeks. HCP: High crude protein; MCP: Medium crude protein; LCP: Low crude protein. (L = Linear effect if *P* ≤ 0.05; Q = Quadratic effect if *P* ≤ 0.05)

Final BW increased linearly with dietary CP level (*P* = 0.003), rising from 529.7 kg in the LCP diet to 550.9 kg in MCP and 564.1 kg in HCP (Table 3). The ADG followed the same pattern (*P* < 0.001), and DMI also increased linearly (*P* < 0.001), with bulls consuming 8.58 kg/d on LCP, 9.90 kg/d on MCP, and 10.21 kg/d on HCP. Carcass traits responded similarly to increasing CP levels. Hot carcass weight increased from 289.3 kg (LCP) to 300.6 kg (MCP) and 312.0 kg (HCP) (*P* = 0.002), while cold carcass weight increased from 287.6 to 298.9 to 310.2 kg, respectively (*P* = 0.002). Carcass yield (*P* = 0.089) and cooling shrinkage (*P* = 0.843) were similar among treatments. A significant treatment × time interaction was observed for DMI across weeks (*P* < 0.001; Fig. 1).

**Table 3:** Effect of treatments on growth performance and carcass characteristic of Nellore bulls

Variables	Treatment^1^				SEM	*P*-Valor		
	HCP	MCP		LCP		Treatment	Linear	Quadratic
Growth performance								
Initial body weight (BW; kg)	384.4	387.41		386.75	1.663	0.443	0.347	0.395
Final BW (kg)	564.13	550.96		529.73	5.638	0.008	0.003	0.575
Dry matter intake (kg)	10.21	9.90		8.58	0.212	0.001	< 0.001	0.087
Dry matter intake (%BW)	2.16	2.11		1.87	0.034	< 0.001	< 0.001	0.050
Average daily gain (kg)	1.69	1.54		1.35	0.046	< 0.001	< 0.001	0.707
Feed efficiency	0.166	0.156		0.157	0.003	0.035	0.079	0.171
Carcass								
Hot carcass weight (kg)	311.97	300.55		289.30	3.483	0.006	0.002	0.985
Cold carcass weight (kg)	310.17	298.89		287.59	3.515	0.006	0.002	0.998
Cooling loss (%)	0.58	0.56		0.60	0.049	0.843	0.867	0.588
Carcass yield (%)	55.31	54.60		54.63	0.002	0.089	0.062	0.203
ADG carcass (kg)	1.13	1.01		0.91	0.028	< 0.001	< 0.001	0.778

¹ HCP: High Crude Protein; MCP: Medium Crude Protein; LCP: Low Crude Protein. BW: Body Weight; ADG: Average daily gain

The NASEM-predicted DMI values were similar among treatments (8.87, 8.88, and 8.84 kg/d for LCP, MCP, and HCP, respectively; *P* = 0.558), showing no linear effect of dietary CP level. In contrast, observed DMI increased linearly (*P* < 0.001), rising from 8.58 kg/d (LCP) to 9.90 kg/d (MCP) and 10.21 kg/d (HCP) (Table 4). Observed net energy for maintenance (NEm) and gain (NEg) also did not differ among treatments (*P* = 0.809 and *P* = 0.804, respectively). Correspondingly, the ratios of observed-to-expected NEm (1.026–1.038; *P* = 0.772) and NEg (1.032–1.044; *P* = 0.861) were statistically similar across treatments. The ratio of observed-to-expected DMI (O: E DMI) showed a clear linear increase (*P* = 0.003), ranging from 0.967 in LCP to 1.114 in MCP and 1.157 in HCP, indicating that steers consuming higher CP diets achieved greater intakes than predicted by the model.

**Table 4:** Effect of treatments in the dry matter intake (DMI), net maintenance (m) energy and aggregate gains (g) and their comparisons with estimated (E) and observed (O) values in Nellore bulls

Variables	Treatment^1^			SEM	*P*-Valor		
	HCP	MCP	LCP		Treatment	Linear	Quadratic
Observed DMI (kg)	10.21	9.90	8.58	0.212	0.001	< 0.001	0.087
Estimated DMI (kg)	8.84	8.88	8.87	0,028	0,558	0.476	0.428
Observed Energy							
NEm (Mcal/kg DM)	2.028	2.012	2.034	0.024	0.809	0.866	0.542
NEg (Mcal/kg DM)	1.368	1.354	1.374	0.022	0.804	0.850	0.540
Observed: Expected							
DMI O: E	1.157	1.114	0.967	0.023	0,008	0,003	0,096
NEm O: E	1.036	1.026	1.038	0.012	0.772	0.912	0.491
NEg O: E	1.044	1.032	1.040	0.015	0.861	0.861	0.617

^1^ HCP: High Crude Protein; MCP: Medium Crude Protein; LCP: Low Crude Protein. The expected energy values (NEm and NEg) are presented in Table 1

## Discussion

The most notable difference between field data and NASEM (2016) estimations was DMI, with observed DMI being higher than predicted in the HCP and MCP treatments. The DMI was estimated using Shrunk Body Weight (SBW) and Net Energy for Maintenance (NEₘ) using NASEM (2016). Despite SBW and NEₘ being similar across treatments, soybean meal was replaced with cracked corn in the LCP treatment, increasing starch and NDF levels compared to other treatments. We speculated that this change led to a faster fulfillment of energy requirements in LCP-fed animals, which reduced DMI in LCP treatment. In the same line, the BRCORTE model estimated that soybean meal had 10% lower TDN than cracked corn. Since NEₘ can be derived from TDN (NASEM 2016), this difference may have influenced intake predictions.

Increasing dietary crude protein is commonly associated with greater DMI in feedlot cattle, particularly when low CP diets restrict rumen nitrogen supply. Adequate rumen-degradable protein enhances microbial growth and fiber fermentation, increases ruminal passage rate, and stimulates voluntary feed intake (Owens and Basalan 2016; NASEM, 2016). In the present study, the linear increase in DMI as dietary CP increased from 11% to 14% aligns with these physiological mechanisms and highlights the role of adequate nitrogen availability in supporting ruminal function and intake regulation.

Diets with lower CP reduced nitrogen availability for microbial growth with reduced DMI, which was shown by Sitorski et al. (2019), We speculated that low supply of dietary N limits ruminal fermentation, which reduced the passage rate of the digesta, resulting in decreased feed intake in diets with lower CP. This response may also indicate greater microbial activity in diets with higher CP, which could contribute to a faster passage of digesta. For optimal microbial activity, a balance between carbohydrate availability and NH₃ concentration is required, as highlighted by Zhang et al. (2020).

Starch is characterized as a rapidly fermentable carbohydrate, as described by NASEM (2016), and its inclusion in the diet enhances NH₃ capture by ruminal bacteria, helping to balance energy and protein supply. Considering that the LCP treatment diet had lower RDP availability and higher starch concentration, we speculated that there was a nutritional imbalance, with insufficient nitrogen supply relative to the energy level of the diet.

Importantly, the predictive equations for intake used by the NASEM model are largely derived from research conducted with Bos taurus cattle raised in temperate climates (Galyean et al. 2011; Anele et al. 2014). These data sets include few animals raised in tropical environments or a representative number of Zebu cattle. This limitation may partly explain the discrepancy between observed and predicted DMI in the present study.

Another factor influencing this divergence is the variation in dietary starch concentrations and the characteristic lower sensitivity of Nellore cattle to high-starch diets (Pacheco et al. 2012; Millen et al. 2015). Starch concentration varied by approximately 14% between treatments, ranging from 576.5 g/kg in the LCP diet to 505.6 g/kg in the HCP diet, showing an inverse relationship with DMI. Compared with taurine cattle, Zebu breeds typically exhibit a reduced tolerance for high-starch diets, primarily due to their greater susceptibility to ruminal acidosis, which can depress intake (Pacheco et al. 2012; Millen et al. 2015). Additionally, greater dietary starch supply increases ruminal propionate production, a known satiety signal. Rathert-Williams et al. (2023), for example, demonstrated that propionate supplementation reduced DMI in steers fed high-grain diets by altering feeding behavior independent of palatability. Thus, the reduction in intake observed with increasing starch concentration in our study is likely the result of increased risk of ruminal acidosis or/and the satiety inducing effects of propionate. This interpretation is further supported by intake patterns throughout the trial (Fig. 1), where the LCP treatment showed lower DMI, mainly in the beginning of the trial, indicating a possible interaction between starch loads, metabolic feedback, and feeding behavior. When DMI was adjusted in NASEM (2016), the predicted performance variables aligned with observed field data, demonstrating that weight gain is heavily influenced by intake. These findings confirm that the predictive equations for performance in NASEM (2016) are highly accurate, provided that DMI is correctly estimated. DMI is the primary determinant of BW gain, as it dictates nutrient intake for tissue development (Davis et al. 2014). Given the NASEM (2016) intake model accounts for similar initial body weights and diet energy levels (Galyean et al. 2011; Anele et al. 2014), only minor differences in DMI between treatments were expected. When comparing observed vs. expected dietary energy values, no significant differences were found between treatments, though all treatments showed higher values than predicted.

Examining the intake curve revealed that by the end of the feeding period, DMI as a percentage of body weight was similar across treatments. As mammals approach their mature BW, adipose deposition increases, a desirable outcome for the meat industry due to its impact on carcass quality and preservation post-slaughter. Leptin, primarily produced by adipocytes, regulates feed intake, and its secretion increases with fat accumulation (Geary et al. 2003). The intake curve during the trial showed that treatments with initially higher DMI experienced a decline after week 10, aligning with the lower-intake treatment. This suggests that animals with greater final BW had higher fat deposition, leading to increased leptin secretion and reduced intake toward the end of the trial. In contrast, the LCP treatment, which had consistently low intake, did not experience this drop, likely due to lower fat deposition and, consequently, lower leptin release. Supporting this, there was a linear trend in carcass yield, with heavier carcasses showing higher yields, reinforcing the link between fat deposition and carcass yield (Dirceu-Pazdiora et al. 2017).

Although metabolizable protein (MP) values were similar across diets, RDP levels differed, with a 10% reduction in HCP vs. MCP or MCP vs. LCP and a 20% reduction in HCP vs. LCP. Wagner et al. (2010) found that in diets containing ~ 80% corn, RDP levels between 5% and 9% of DM influenced DMI linearly while maintaining a constant supply of rumen-undegradable protein (RUP), directly impacting daily weight gain. Similarly, Sitorski et al. (2019) reported a quadratic effect on DMI in taurine cattle when RDP ranged from 3.02% to 9.85% of DM, with maximum intake at 8.91% RDP. However, neither study evaluated Zebu cattle. In contrast, studies on Zebu cattle with RDP levels similar to those in this trial found no significant differences in DMI (Amaral et al. 2018; Menezes et al. 2016). The limited sample sizes in previous studies make precise evaluations of DMI variations more challenging.

In this study, digestibility exhibited a linear response across all evaluated parameters, inversely to DMI. Bull that showed lower DMI had higher nutrient digestibility, likely due to a reduced passage rate, which allows for prolonged microbial exposure and improved nutrient utilization (Van Soest 1994). This effect is well-documented in ruminant nutrition, where lower intake often leads to increased retention time in the rumen, enhancing fiber degradation and microbial efficiency (Hall et al. 2015). Additionally, Forbes (2007) highlighted that as passage rate decreases, ruminal fermentation improves, leading to greater volatile fatty acid production and energy utilization.

Increasing DMI was the primary driver of weight gain in this study, as it provides the essential nutrients required for tissue development; consequently, the HCP group achieved higher hot and cold carcass weight through greater. This is supported by the linear trend in carcass yield, where heavier carcasses from high-protein treatments showed higher weight, reinforcing the link between adequate protein supply, increased weight accumulation, and improved commercial value for the meat industry. In contrast, cooling loss and carcass yield were not affected by treatments, showing that the treatment effect was more concentrated in increase body weight.

## Conclusion

Advancements in protein requirement modeling for cattle have been significant, with MP emerging as the most accurate predictor of animal responses to specific diets. However, this study indicates that the NASEM model underestimates DMI for Nellore cattle in diets with high protein concentration. This variation in intake may be influenced by factors beyond MP and dietary NEg. From a practical point, this underestimation may lead to inaccuracies in diet formulation, potentially resulting in over- or under-supply of nutrients. Nutritionists working with Nellore cattle, especially under tropical conditions, should therefore interpret model outputs with caution and consider adjusting predicted intake values based on observed performance. Additionally, diets with high starch concentrations resulted in reduced DMI and, consequently, reduced DMI compared with diet with 14% of CP from soybean meal.

## Data Availability

The data sets generated during and/or analyzed during the current study are available from the corresponding author on reasonable request.
